# Roles of Elongator Dependent tRNA Modification Pathways in Neurodegeneration and Cancer

**DOI:** 10.3390/genes10010019

**Published:** 2018-12-28

**Authors:** Harmen Hawer, Alexander Hammermeister, Keerthiraju Ethiraju Ravichandran, Sebastian Glatt, Raffael Schaffrath, Roland Klassen

**Affiliations:** 1 Institut für Biologie, FG Mikrobiologie, Universität Kassel, Heirich-Plett-Str. 40, 34132 Kassel, Germany; harmenhawer@uni-kassel.de (H.H.); alex_hammermeister@t-online.de (A.H.); 2 Max Planck Research Group at the Malopolska Centre of Biotechnology, Jagiellonian University, 30-387 Krakow, Poland; er.keerthiraju@gmail.com (K.E.R.); sebastian.glatt@uj.edu.pl (S.G.); 3 Postgraduate School of Molecular Medicine, 02-091 Warsaw, Poland

**Keywords:** epitranscriptomics, tRNA, tRNA modification, Elongator, wobble uridine modifications, U_34_, diphthamide, neurodegeneration, cancer

## Abstract

Transfer RNA (tRNA) is subject to a multitude of posttranscriptional modifications which can profoundly impact its functionality as the essential adaptor molecule in messenger RNA (mRNA) translation. Therefore, dynamic regulation of tRNA modification in response to environmental changes can tune the efficiency of gene expression in concert with the emerging epitranscriptomic mRNA regulators. Several of the tRNA modifications are required to prevent human diseases and are particularly important for proper development and generation of neurons. In addition to the positive role of different tRNA modifications in prevention of neurodegeneration, certain cancer types upregulate tRNA modification genes to sustain cancer cell gene expression and metastasis. Multiple associations of defects in genes encoding subunits of the tRNA modifier complex Elongator with human disease highlight the importance of proper anticodon wobble uridine modifications (xm^5^U_34_) for health. Elongator functionality requires communication with accessory proteins and dynamic phosphorylation, providing regulatory control of its function. Here, we summarized recent insights into molecular functions of the complex and the role of Elongator dependent tRNA modification in human disease.

## 1. Epitranscriptomic Transfer RNA Regulation

**Figure 1:**
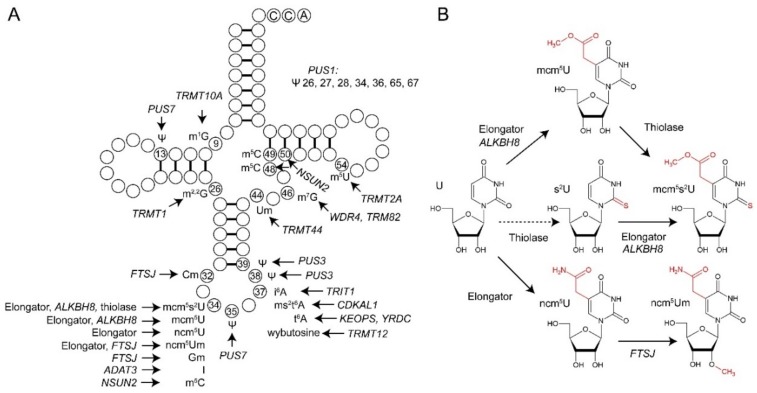
Transfer RNA (tRNA) modifications associated with human disease. (**A**) Schematic representation of a cytoplasmic tRNA with disease linked modifications at indicated base positions. Modification genes linked to human diseases when mutated or upregulated are denoted (see Table 1 and references therein for details). (**B**) Overview of steps and genes involved in xm^5^U_34_ synthesis. A broken line between U_34_ and s^2^U_34_ indicates the fact that several lines of evidence support preferential action of the thiolase on mcm^5^U_34_ rather than unmodified U_34_. Elongator specifies different subunits of the Elongator complex, whereas thiolase represents the complex composed of subunits CTU1 and CTU2. Abbreviations for U_34_ modifications are according to the modomics database [14].

Apart from the four standard nucleosides, cellular RNA contains a broad variety of posttranscriptional modifications which may have significant impact on its function [1]. The total RNA modification set of a cell is termed the epitranscriptome, and its dynamic changes are thought to represent a strategy to modulate RNA function involving “writers” (modifiers), “erasers” (demodifiers) and “readers”, proteins specifically recognizing RNA modifications [2,3]. Among the different types of RNA subject to epitranscriptomic changes, transfer RNA (tRNA) harbors by far the most abundant and chemically diverse modifications (Figure 1). While initially thought to be constitutively modified, numerous examples are known by now demonstrating dynamic changes in tRNA modification patterns in response to changing environments or conditions [4,5,6,7,8,9,10,11,12,13]. One of these involves a writer methylase and an eraser demethylase, as observed in epitranscriptomic regulation of messenger RNA (mRNA) translation [10]. In this case, mammalian ALKBH1 was identified as a demethylase removing the methyl-group in 1-methyladenosine (m^1^A), which is found in initiator and elongator tRNAs and introduced by TRMT6 (substrate binding subunit) and TRMT61 (catalytic subunit) [10]. It was demonstrated that epitranscriptomic modulation of m^1^A presence in both tRNA types (initiator and elongator) regulates tRNA function in translation initiation and elongation in a dynamic manner in response to glucose availability [10]. Importantly, the absence of the eraser protein causes embryonic lethality or neural defects in a mouse model system, indicating a direct or indirect role of epitranscriptomic changes in mammalian tRNA for prevention of disease [10].

Despite the fact that m^1^A represents the only tRNA modification known so far to be actively removed by an eraser protein, a variety of additional modifications are altered in their relative abundance in response to changing environmental or physiological conditions. These triggers include elevated temperature, oxidative stress or availability of nutrients required for synthesis of modifications, e.g., sulfur, bicarbonate, queuine or taurine [4,5,6,9,11,13]. Due to the growing body of evidence for a dynamic, rather than static nature of tRNA modifications, additional examples for epitranscriptomic regulation of tRNA function should be envisioned.

## 2. Disease Related Transfer RNA Modifications

For tRNA to function as the adaptor molecule in translation, its folding into an l-shaped structure is essential [1]. The structure is characterized by tertiary base-pairing and the presence of stems formed by intramolecular base-pairing in addition to single-stranded loops. A number of posttranscriptional modifications in unpaired tRNA loop regions contribute to tRNA structure by preventing canonical base-pairing [15]. Not only cytoplasmic but also mitochondrial tRNA is subject to dynamic modification by enzymes encoded in the nucleus [9,11]. A variety of human diseases are linked to mutations in genes required for introduction of modifications [16,17,18,19,20] in both cytoplasmic and mitochondrial tRNAs (Figure 1 and Table 1). Diseases associated with defects in human tRNA modification genes include various neurological syndromes as well as metabolic and respiratory dysfunctions (Table 1). In addition, an upregulation of different tRNA modification genes is observed in various cancer types (Table 1), suggesting an increased demand for the activity of tRNA modifiers in different tumor cell types.

Among disease-relevant modifications targeting cytoplasmic tRNA species, neurological disorders appear to be most common. These include intellectual disability, familial dysautonomia (FD), epilepsy and other syndromes. Interestingly, various mutations known to cause defects in different tRNA modifications such as 5-methylcytosine (m^5^C), 5-methoxycarbonylmethluridine (mcm ^5^U), 5-methoxycarbonylmethyl-2-thiouridine (mcm^5^s^2^U), 5-carbamoylmethyluridine (ncm^5^U), pseudouridine (Ψ) at positions 38/39 and 13/35, 1-methylguanosine (m^1^G), inosine (I) and N2,N2-dimethylguanosine (m^2,2^G) are all associated with intellectual disability in humans (Table 1), suggesting normal brain function and development to be particularly dependent on the presence of these modifications.

**Figure 2:**
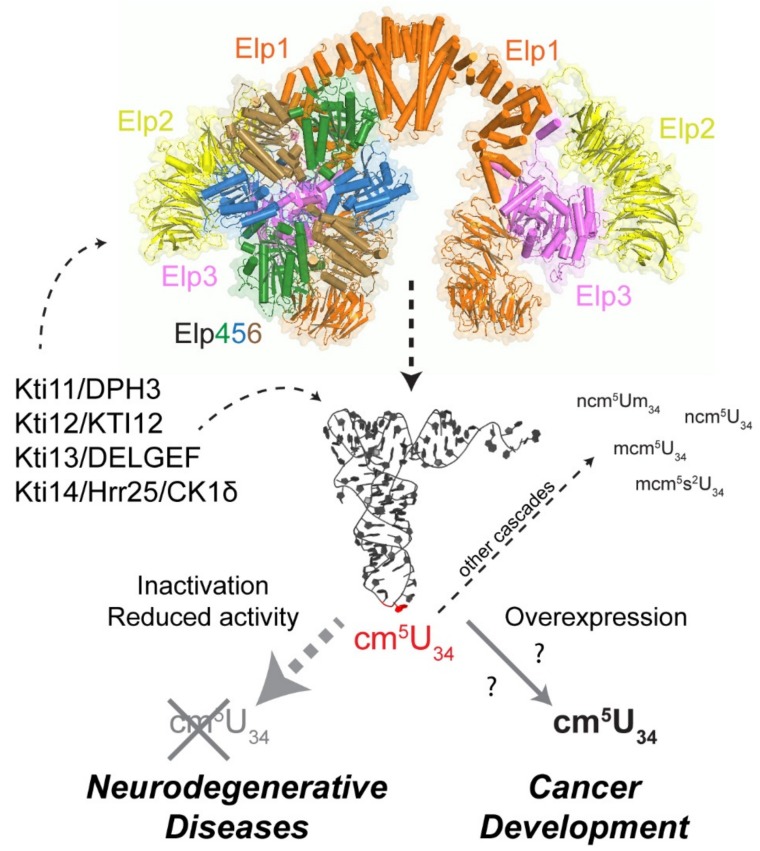
U_34_ modifications in human diseases. Scheme showing the “Janus headed” nature of the Elongator complex that plays an important role in human health and disease. (Top) The pseudo-atomic model of the fully assembled Elongator complex is shown in cartoon and transparent surface representation (Elp1/orange, Elp2/yellow, Elp3/pink, Elp4/green, Elp5/blue, Elp6/brown). Additional regulatory factors (Kti11-Kti14, left) and subsequent modifications (right) are indicated and labeled (for further details, see text). (Bottom) The opposing roles of reduced or enhanced levels of cm^5^ modifications in neurodegenerative diseases and cancer are highlighted.

Considering the position of critical modifications within the tRNA (Figure 1), a remarkable feature is that many of the disease-relevant tRNA modifications are naturally found within the anticodon loop and more specifically, at the wobble position (34) of the anticodon. Wobble base modification defects in mitochondrial tRNAs are also linked to various myopathies and may also induce neurological symptoms (Table 1) [19,20,37], highlighting the central role of wobble base modifications for normal functioning of tRNA in the different cellular compartments. Modifications at the wobble position are known to enable expanded base-pairing possibilities (inosine) [68] or to improve translational fidelity and elongation by optimizing codon translation rates (mcm^5^s^2^U) [69,70,71,72,73,74]. Wobble uridine modifications of the xm^5^U type (mcm^5^U, mcm^5^s^2^U and ncm^5^U; Figure 1B) belong to the most complex tRNA modifications in terms of involved genes (Table 2) and pathways as well as with respect to posttranslational modification of the tRNA modifier complexes themselves [66]. Multiple correlations of xm^5^U defects (Table 1) with human disease indicate this modification family to be of outstanding relevance for human health and development (Figure 2).

## 3. Elongator Dependent Transfer RNA Modifications

In anticodons of a subset of tRNAs, modifications of wobble uridines (U_34_) require the activity of the Elongator complex (for recent reviews, see [18,75,76]). Target tRNAs receiving Elongator dependent modifications are tRNA^Lys^_UUU_, tRNA^Gln^_UUG_, tRNA^Glu^_UUC_, tRNA^Arg^_UCU_, tRNA^Gly^_UCC_, tRNA^Ser^_UGA_, tRNA^Pro^_UGG_, tRNA^Thr^_UGU_, tRNA^Ala^_UGC_ and tRNA^Leu^_UAA_ [77,78]. Elongator is a multi-subunit (Elp1–Elp6) protein assembly that was originally isolated from yeast in association with elongating RNA polymerase II holo-enzyme [79,80] and hence implicated in mRNA transcription rather than translation [78,81,82].

However, Elongator is now known to assemble into an active holo-dodecameric complex [(Elp1–Elp6)_2_] that binds tRNAs with different subunits [83,84,85,86,87,88]. Accordingly, Elongator operates as a tRNA modifier in a conserved pathway that is found in all three domains of life and shown to be functionally exchangeable between eukaryotic model organisms [86,89,90,91,92,93]. In yeast, the complex attaches to U_34_ from eleven different tRNA species 5-carboxy-methyl (cm^5^) groups [77,78], which in concert with additional enzymatic U_34_ modifier cascades can be further converted to even more composite modifications including (but not limited to) ncm^5^, mcm^5^ or mcm^5^s^2^ (see Figure 1B, [77,78]). Formation of the cm^5^ side chain is directly catalyzed by Elp3, Elongator’s catalytic core subunit, which carries binding domains for tRNA and acetyl-CoA and a radical SAM (S-adenosyl-methionine) motif that coordinates an organometallic [4Fe–4S]_SAM_ cluster shown to be crucial for U_34_ modification in vivo [78,81,89,94]. In support of this notion, archaeal Elp3 from *Methanocaldococcus infernus* has been shown to modify U_34_ in the anticodon loop of synthetic tRNA^Arg^_UCU_ substrates iin vitro in a radical SAM dependent reaction containing electron donor (Na_2_S_2_O_4_), SAM and acetyl-CoA [93]. Moreover, sophisticated insights into the basis and mechanism for Elongator’s tRNA modification capacity have been recently provided through elegant structure-functional work on prokaryotic Elp3 from *Dehalococcoides mccartyi* and holo-Elongator from the yeast *Saccharomyces cerevisiae* [86,87,88,95,96,97].

In addition to the biochemical isolation of Elongator (see above), genetic screens aimed at isolating yeast mutants that resist anticodon cleavage by zymocin, a U_34_ modification-dependent tRNase ribotoxin, were also instrumental in identifying Elongator subunits and accessory proteins with roles related to U_34_ modification and regulation [78,98,99,100,101,102,103,104]. Together with other strategies from many independent research labs, these approaches collectively led to the identification of the U_34_ wobble methylase complex (Trm9•Trm112) [101,105,106,107] and components of a pathway (Nfs1, Tum1, Uba4, Urm1, Ncs2, Ncs6) for Elongator related U_34_ thiolation [99,101,108,109,110,111,112,113,114,115,116]. The methyltransferase (MTase) activity of Trm9•Trm112 appears to depend on Elongator activity for the concerted formation of mcm^5^ and mcm^5^s^2^ in yeast and related modifications by homologs of wobble methylase (ALKBH8•TRMT112) in higher eukaryotic systems [117,118,119,120]. Intriguingly, Trm112 not only promotes the MTase activity of the catalytic Trm9 subunit but also acts as an activating hub or platform for three more SAM-dependent MTases modifying ribosomal RNA (Bud23), tRNA (Trm11) or eRF1 translation termination factor (Mtq2) [121,122]. Collectively, this illustrates the importance of methyltransferases for mRNA translation and *de novo* protein biosynthesis.

As for U_34_ thiolation and formation of the mcm^5^s^2^ modification, it has been shown that S-incorporation into U_34_ substrate tRNAs by yeast wobble thiolase (Ncs2•Ncs6 aka CTU1•CTU2, see Figure 1) requires a conserved S-relay system (Nfs1, Tum1, Uba4) for S-activation, S-mobilization and S-transfer onto Urm1, a ubiquitin related modifier protein [109,110,111,112,113,114]. Urm1 is unique in combining features typical of prokaryotic S-carriers with eukaryotic ubiquitin proteins [123,124]. In line with this notion, Urm1 can act as a non-canonical, lysine-directed protein modifier in a pathway known as protein urmylation and as a sulfur donor for U_34_ thiolation [112,113,114,125]. Both roles are strictly sulfur-dependent and exchangeable among eukaryotes [115,116,126] and based on Urm1-like proteins from archaea and bacteria [127,128]; they appear to be conserved and important in all domains of life. In line with this, their inactivation triggers stress-induced growth defects in microbes, organ underdevelopment in plants and, strikingly, lethality in flies [108,113,124,129,130,131]. In contrast to ubiquitin activation and conjugation by conventional E1-E2-E3 enzyme cascades, no E2/E3 activities for Urm1 are known to this end, and Urm1 activation by its E1-like enzyme Uba4 results in C-terminal thiocarboxylation (Urm1-COSH) that is crucial for both urmylation and tRNA thiolation [113,115,123,132,133].

The formation of Urm1-COSH is very similar if not identical to E1-like (MoeB or ThiF) activation of bacterial S-carrier proteins (MoaD or ThiS) that (rather than being involved in protein conjugation) solely donate sulfur for synthesis of molybdopterin or thiamine co-factors [134,135]. Thus, apart from its similarity to eukaryotic ubiquitin-like proteins, Urm1 indeed relates to prokaryotic S-carrier proteins, which is why the protein was coined a molecular fossil at the cross-road of protein and RNA modifications [123]. Its dual-functionality requires desulfurase Nfs1, which mobilizes sulfur from cysteine for direct S-transfer onto Uba4 [110,111] or indirectly via sulfur transferase Tum1 [109,112,113,114]. Uba4 is equipped with MoeB-like (MoeBD, see above) and rhodanese-type domains (RHD) that carry thiol-active cysteines [113,133]. S-transfer to the one in RHD results in a persulfide, which, following adenylation of Urm1 by the MoeBD, likely forms an acyl-disulfide with the Urm1 modifier [132,133,136]. Upon reductive cleavage of this bond, Urm1-COSH gets released [137] to be able to operate in urmylation or donate the activated sulfur species for S-insertion into tRNAs by thiolase Ncs2•Ncs6 [115,133]. Recently, in eukaryotic Ncs6 and TtuA (a related thiouridine synthetase from thermophilic bacteria and archaea) [3Fe-4S] and [4Fe-4S] clusters were identified, respectively, that appear to be involved (directly or indirectly) in the thio-modification reaction required for s^2^U formation.

Together with findings that tRNA thiolation is apparently not required for protein urmylation and vice versa, the two *URM1* pathway branches—albeit mechanistically linked through sulfur activation—seem to be functionally separated from rather than dependent on each other [115]. Thus, a previously suggested concept, according to which the sulfur flow from Urm1-COSH to tRNA thiolation may be kept in-check by urmylation [138], seems less likely to date. However, in this context, it is noteworthy that human URM1 (hURM1) and Urm1-like proteins (SAMP, TtuB) have been shown to form urmylated conjugates with human and prokaryotic orthologs of yeast thiolase (CTU1, CTU2, NcsA, TtuA) [125,127,128]. Whether or not this implies that S-transfer (via Urm1-COSH) for tRNA thiolation may involve direct urmylation of thiolase subunits is unknown but attractive to support the option of interdependence among the two *URM1* pathway branches, protein urmylation and U_34_ thiolation. Although the S-donor role of Urm1 for tRNA thiolation has been demonstrated iin vitro [137], we are not aware of sulfur transfer during lysine-directed urmylation of protein targets (including thiolase components, see above) in yeast or other model organisms. Nonetheless, it has been shown in human cell lines that IKAP (the homolog of yeast Elongator subunit Elp1) was urmylated under conditions of oxidative stress [116]. Additional urmylation target proteins, including human thiolase subunits CTU1 and CTU2 (see above) have been suggested as well [125]. Since the relevance of IKAP urmylation for Elongator’s U_34_ modification function in human cells has not been addressed and a functional link (if any) between urmylation of IKAP/Elp1 and thiolase (CTU1•CTU2) is not clear for the time being, these intriguing phenomena remain to be resolved in the future.

## 4. U_34_ Modifications and Neurodegeneration

Notably, neurons are known to be particularly sensitive to translational defects for some time [139]; however, it was only recently shown that tRNA modifications in combination with the specific usage of AA-ending over AG-ending codons fine tune the translation of specific neuronal transcripts [140]. In addition, a single point mutation in the Elongator subunit Elp6 was identified in a novel cerebellar ataxia mouse model that triggers the cell type specific degeneration of Purkinje neurons [141]. The neuronal decay is accompanied by proteotoxic stress and substantial microgliosis, which can be partially delayed by blocking the NLRP3 inflammasome. The Elp6 mutation leads to a destabilization of the Elp456 subcomplex and reduces the tRNA modification levels [141] in a similar extent as FD patients carrying Elp1 mutations (Table 1) [21].

These similarities indicate that a certain reduction of modification levels causes severe cellular malfunctions, whereas more dramatic alterations would not permit the survival of the patients. Additionally, patients of sporadic amyotrophic lateral sclerosis (ALS) show reduced levels of Elp3 protein and mcm^5^s^2^U in the motor cortex [27]. The authors of this study further demonstrated that the role of Elp3 in the pathogenesis of ALS is mediated through its tRNA modification activity, which provided yet another link between tRNA modification defects and neurodegeneration. However, the study surprisingly reported that the SAM domain of Elp3 seemed sufficient to rescue the effects. This observation is in stark contrast to biochemical [86,93] and functional [69,89] studies, showing that both the SAM and acetyl-CoA binding domains are necessary for Elp3’s tRNA modification activity (see above). In summary, the list of neurodegenerative diseases that are directly and indirectly connected to the disruption of the Elongator tRNA modifier complex is continuously growing.

## 5. A Role of Protein Aggregation in Neurodegeneration

Using ribosome profiling, the absence of mcm^5^s^2^U in yeast tRNA^Gln^_UUG_ and tRNA^Lys^_UUU_ was shown to result in a translational slow down at cognate glutamine and lysine codons [70]. This defect goes hand-in-hand with the accumulation of cellular protein aggregates [70]. While the direct mechanism of aggregate formation remains to be solved, a striking observation was that a large overlap exists between aggregates induced by tRNA hypomodification and those formed in the absence of a functional ribosome associated chaperone complex (*ssb1/ssb2*) [70,142]. This may indicate that ribosomal slow down perturbs folding of the nascent polypeptides. Genetic approaches in budding yeast further support that formation of protein aggregates in the absence of different important tRNA modifications represents a key trigger of pleiotropic cellular defects in cell polarity, morphogenesis and nuclear segregation during cell division [143].

Importantly, protein aggregation in the absence of U_34_ modification is not limited to the yeast model system but was also observed in nematodes, mice and human cells [27,32,33,70,144,145]. In addition, the absence of the mitochondria-specific taurine-derived wobble uridine modifications induce aggregation of mistargeted mitochondrial proteins [146]. Interestingly, protein aggregation is also a hallmark of various neurodegenerative diseases, and therefore, might represent a functional link between tRNA modification defects (affecting mcm^5^s^2^U and other modifications) and neurodegenerative or neurodevelopmental diseases. In support of this, it was already demonstrated that a conditional *ELP3* knockout in mice induced the unfolded protein response (UPR) and caused reduced numbers of cortical projection neurons, leading to neurodevelopmental defects and microcephaly [144]. While UPR induction in tRNA modification mutants seems to be confined to higher eukaryotic cell systems, aggregation of endogenous cytoplasmic proteins is observed in both mammals and yeast cells [27,32,33,70,144,145,147].

Since mutations in *ELP3* are also linked to the fatal degenerative motor neuron disorder ALS (see above and Table 1), protein aggregation in a mouse motor neuron-like cell line was also analyzed. Upon depletion of *ELP3* from this cell line, protein aggregation including the ALS relevant mutant form of SOD1 was observed [27]. In addition, several lines of evidence indicated the neurodegenerative effects of the ELP6 mutation causing ataxia-like syndromes in a mouse model (see above) to be accompanied by protein aggregation [141]. Additionally, silencing of either *ELP3* or *CTU2* (s ^2^U) in human melanoma cells resulted in the induction of endogenous protein aggregates [32], suggesting the effect of Elongator and tRNA thiolation defects on protein solubility to be general and highly likely disease relevant. In the latter case, it was also observed that a key factor reprogramming cancer cell gene expression (see below) depends on wobble uridine modification for efficient translation and is present in the endogenous aggregates [32]. This observation is consistent with the proposal that reduced codon translation rates in the absence of mcm^5^s^2^U cause protein folding defects and aggregation of the nascent polypeptide. The finding that protein aggregation may also be induced in yeast by defects in other modifications, such as Ψ_38/39_ or t^6^A [143,148] and that the very same defects in human cells cause related neurodegenerative/neurodevelopmental syndromes [41,47,60] provide solid support for the assumption that protein aggregation may represent an important trigger of neurodegenerative disease correlated with mutations in tRNA modifiers.

## 6. U_34_ Modification in Cancer

In addition to their roles in prevention of neurodegeneration, enzymes introducing U_34_ tRNA modifications were also identified as key factors to sustain metastasis of breast and bladder cancer and survival of malignant melanoma cells [32,33,149]. It has been known for some time that genes in DNA damage repair pathways display a certain codon-bias [130,150] and that loss of Elongator or related pathways (e.g., Uba4,Urm1, see above) can induce DNA damage [151]. This knowledge has recently been extended by showing that the very same pathways are crucial determinants for the survival of therapy-resistant melanoma cells. In detail, *BRAF^V600E^* is the most prevalent mutation among human melanoma patients and responsible for resistance to targeted therapy. ELP1, ELP3, CTU1 and CTU2 are strongly upregulated in *BRAF^V600E^* cells and inactivation of *ELP3* impaired the development of *BRAF^V600E^* melanoma in a zebrafish model [32]. Moreover, it was shown that the Elongator complex promotes glycolysis in melanoma cells through direct, codon-dependent, regulation of the translation of Hypoxia Induced factor 1 α (*HIF1A)* mRNA, and the maintenance of high levels of HIF1α protein providing strong resistance to anti-BRAF therapy [32]. In a previous study the migration and tumorigenicity of melanoma-derived cells was shown to be significantly decreased upon depletion of ELP1, ELP3, ELP5 or ELP6 in melanoma cells [31]. In another study, ELP3 was shown to be upregulated in human hepatocellular carcinoma (HCC) cells, which correlated well with the phosphorylation of protein kinase B (AKT) [152]. The Elongator mediated migration and invasion of HCC cells is further promoted by the induced expression of MMP-2 and MMP-9 through the PI3K (phosphoinositide 3-kinase)/AKT signaling pathway. ELP3 was also shown to drive Wnt-dependent tumor initiation and regeneration in the intestine by maintaining a subpopulation of cells expressing LGR5- and SOX9 cells [153]. Furthermore, genetic ablation of ELP3 strongly impaired invasion and metastasis formation in a model system of invasive breast cancer. In detail, ELP3 and CTU1•CTU2 are upregulated in human invasive breast cancer and support cellular invasion through the translation of the DEK oncoprotein, which subsequently promotes the IRES (internal ribosome entry site)-dependent translation of the pro-invasive transcription factor LEF1 [33]. In addition, ALKBH8, the methyltransferase implicated in the final step of mcm^5^s^2^U and mcm^5^U formation (Figure 1) is highly expressed in bladder cancer and *ALHBH8* knockdown induces cancer cell death due to reduced expression of the anti-apoptotic protein survivin [29,149].

Hence, multiple lines of evidence indicate U_34_ modifications to promote cancer cell growth and metastasis by ensuring efficient translation reprograming upon transition from normal to cancer cell growth mode. In addition to upregulation of Elongator, *ALKBH8* and thiolase genes *CTU1* and *CTU2* genes, other tRNA modification genes, including *NSUN2* (m^5^C) and *METTL1* (m ^7^G) also become overexpressed in human cancers [52,54,154,155] (Table 1). Moreover, the latter two genes are implicated in resistance against anti-cancer therapy [156] since *NSUN2* upregulation is correlated with poor prognosis in patients with in Head and Neck Squamous Carcinoma [157]. Changes in epitranscriptomic tRNA modification in cancer cells may have a general broad significance for prognosis of disease progression and therapy. Hence, a greater understanding of the underlying pathways involved in the modification of U_34_ is necessary to further understand its two-faced character in human diseases. It remains to be shown if a specific inhibitor for Elongator can be identified and developed. First and foremost, a targeted therapy against Elongator must define a therapeutic window that permits the treatment of cancer cells and simultaneously avoids negative effects on neuronal tissues.

## 7. Phosphoregulation of Elongator Involving Kti12 and Kti14/Hrr25

Several studies in yeast and other model organisms have shown that tRNA modifications, including Elongator dependent ones can change in response to cell cycle progression and different environmental stresses. This indicates that tRNA modifications are subject to regulation rather than being constitutively formed [158,159,160]. In case of the Elongator complex from yeast, several accessory proteins have been described that influence its tRNA modification activity, namely Kti11-Kti14/Hrr25 and Sit4 (Figure 2). Consistent with this, a casein kinase 1 (CK1) isozyme (Kti14/Hrr25), type 2A protein phosphatases (Sit4·Sap185 and Sit4·Sap190) and an Elongator interactor (Kti12) were shown to affect the phosphorylation state of Elongator’s largest scaffold subunit Elp1 [98,161,162,163]. In principle, dynamic phosphorylation may have an impact on Elongator’s catalytic Elp3 subunit, its localization or its ability to interact with substrate tRNAs [164,165,166]. Although it is unclear what precise role Elp1 phosphorylation plays, it has been proposed as an ‘on/off’ switch for Elongator’s U_34_ modifier activity, for example, in response to growth conditions or cellular stress. Given that translation of some mRNAs are indeed dependent on proper U_34_ modification, and hence tunable by Elongator [130,167,168], and that tRNA modifications including Elongator-dependent ones do oscillate, this raises the option that Elongator is part of a translational control mechanism which functions through its role as a U_34_ modifier. Such a role, which reflects that Elp1 phosphorylation by casein kinase Kti14/Hrr25 is largely positive for Elongator’s performance, is consistent with loss-of-function phenotypes associated with kinase-dead *hrr25/kti14* mutations, ablative Elp1 phosphosite substitutions and specific inhibition by ATP analogs of an analog-sensitive Hrr25 kinase variant (Hrr25-I82G) [164,165]. Although Hrr25 operates on many cellular functions [169], which complicates providing clear insights into Elp1 phosphorylation signals, its kinase activity is required for full functionality of ribosomes and U_34_ containing tRNAs demonstrating its importance in mRNA translation and protein synthesis [164,165,170,171,172]. Since dynamic IKAP/Elp1 phosphorylation was also observed in human melanoma cells [32] and change in response to insulin availability, pharmacological interference with this process may represent an attractive option to downregulate the Elongator function and possibly impair cancer cell proliferation.

A key component in the phosphoregulation of Elp1 seems to be the Elongator partner protein Kti12 (Figure 2). Although the precise role of Kti12 is ill-defined, the yeast protein and its plant ortholog (DRL1/ELO4) carry N-terminal P-loop motifs that are typical of nucleotide binding kinases and NTPases [173,174,175,176,177]. Consistent with a functional role for this domain, a P-loop truncation triggers defects typical of Elongator mutants including loss of U_34_ modification [98]. Importantly, Kti12 interacts with the Hrr25 kinase in an Elongator-dependent fashion and in doing so, apparently supports Elp1 phosphorylation [98,100,102,178]. This is based on data showing that *KTI12* gene deletions abolish Elongator interaction with Hrr25, cause U_34_ modification defects typical of *elp* (and *hrr25*) mutants and trigger formation of hypo-phosphorylated Elp1 isoforms similar to kinase-dead *hrr25* cells [101,161,165]. 

## 8. Elongator Regulation via Kti11/Dph3 and Kti13

Two additional factors involved in Elongator regulation are Kti11 (alias Dph3) and Kti13 (alias Ats1) [179] (Figure 2). Kti11 precipitates Elongator subunits (Elp1, Elp2, Elp3, Elp5) and associates with Kti13 [108,180,181] in a hetero-dimer shown not only to affect Elongator’s U_34_ modification activity but also to relate to a posttranslational protein modification pathway [108,180,181,182,183,184]. The Kti11/Dph3•Kti13 complex has been implicated in electron transfer to Elp3 for radical SAM dependent U_34_ modification by the Elongator complex [182,183]. Removal of Kti11 (i.e., no electron flow) was found to eliminate the U_34_ modifier activity of Elongator while loss of Kti13 significantly reduced the tRNA modification function to ~20% of wild-type levels [78,180,181]. Kti11/Dph3 partakes together with Dph1, Dph2, Dph4, Dph5, Dph6 and Dph7 in the synthesis of diphthamide, a posttranslationally modified histidine residue (His699 in yeast; His715 in humans) found on translation elongation factor 2 (EF2) [185,186]. EF2 is an essential translation factor that mediates the translocation of the ribosome on the mRNA during translation elongation. Strikingly, diphthamide modified EF2 is the target for diphtheria toxin (DT), which inactivates the translation factor via ADP (adenosine diphosphate)-ribosylation, and thereby, induces death of the intoxicated cell [187,188,189]. Thus, the absence of Dph3/Kti11 leads to full resistance to the lethal ADP-riboslyase activity of DT [180,182,185,186,190]. These data clearly indicate a pathological role for the diphthamide modification in cell growth and proliferation control. The physiological relevance of diphthamide-modified EF2 is less evident [191,192]. However, recent functional and structural analyses suggest diphthamide-modified EF2 supports reading frame maintenance and reduces ribosomal errors [190,193,194,195,196,197].

Based on structure-function analyses, Kti11/Dph3 carries a metal (iron, zinc) binding domain [182,183,198] that is essential for both the modification of U_34_ carrying tRNAs by Elongator and the synthesis of diphthamide on EF2 [182,183]. Similar to bacterial rubredoxins, Kti11/Dph3 is a redox-active protein and capable of electron transfer to the iron-sulfur clusters of Elongator’s core subunit Elp3 [86,89] and the radical SAM enzyme Dph1•Dph2, which is essential for the first step of diphthamide biosynthesis [108,180,186,199]. Dong et al. (2014) reconstituted a reaction iin vitro, in which Dph3/Kti11 was able to feed electrons into the [4Fe–4S]_SAM_ cluster of the Dph1•Dph2 enzyme for reductive SAM cleavage and generation of a 3-amino-3-carboxypropyl radical (ACP) subsequently used for formation of ACP-EF2, the first intermediate of the diphthamide pathway [200,201,202,203]. Moreover, the Dph3/Kti11 reductase Cbr1 has been identified, which is required for recycling the electron carrier and transfer function of Dph3/Kti11 to both the U_34_ and the diphthamide modification pathways [204].

The formation of a stable Kti11/Dph3•Kti13 heterodimer suggested an involvement of not only Kti11, but also Kti13 in diphthamide formation [181,182,183]. Indeed, a yeast *KTI13* gene deletion strain was shown to confer protection against growth inhibition by DT (see above) [182]. Apart from Kti11/Dph3 and Cbr1, Kti13 may, therefore, be yet another factor operating in both the radical SAM pathways for Elongator dependent tRNA modification and diphthamide synthesis on EF2 [182,183].

Table 1 lists up severe pathologies in humans that are linked with Elongator dependent and related tRNA modification defects. Similarly, defects in genes involved in the diphthamide synthesis pathway of higher eukaryotic model organisms can associate with a variety of diseases and syndromes (Table 3) such as tumorigenesis [205,206,207,208,209,210,211], intellectual disability, craniofacial abnormalities [212,213] and Miller-Dieker Syndrome (MDS) [214], as well as airway obstruction and external genital abnormalities [215] in humans. In mice, embryonic lethality, edema, polaydactyly, jaw shortening, cleft palate, necrosis, apoptosis and defects in placenta development as well as neuronal underdevelopment have been observed in conjunction with diphthamide defects [214,216,219,220]. Furthermore, sensitivity to oxidative stress was reported in chinese hamsters to correlate with failure to diphthamide-modify EF2 [218]. Moreover, failure of intestinal stem cell proliferation has been strictly associated with malfunctional Dph1 and Dph5 in *Drosophila melanogaster* [217]. Hence, defects in both the radical SAM pathways for tRNA and EF2 modifications, which have been shown to interfere with the fidelity and efficiency of mRNA translation elongation and trigger ribosomal errors, are potentially linked to a multitude of diseases (Table 1 and 3) from several eukaryotes. With the Kti11/Dph3•Kti13 heterodimer and the Cbr1 reductase qualifying as prime candidates to interconnect and possibly, cross-link U_34_ and diphthamide modifications, there is the emerging prospect for regulation of disease related radical SAM modifier enzymes by a common mechanism that involves control by electron flow to (and from) their iron-sulfur centers. Further studies will be required to address whether drug-based interference with Kti11/Dph3•Kti13 and/or Cbr1 functioning may provide new intervention schemes against disease syndromes linked with dynamic changes of epitranscriptomic tRNA modifications typical of defects in Elongator or the related diphthamide synthesis pathway.

## 9. Conclusions

tRNA modifications are required for optimal translational efficiency and fidelity. A growing list of dynamic tRNA modifications suggests a role in adaptation of translational efficiency to changes in environmental conditions. Consequently, several of these modifications are necessary to prevent human disease. Among the diseases linked to modification/modifier defects, neurodevelopmental syndromes are common, pointing to a specific requirement of full tRNA modification sets in neuronal cells. Among the different modifications linked to human disease, wobble uridine modifications dependent on the Elongator complex are prevalent. Elongator dependent tRNA modifications are not only required for human brain cell function, but also for cancer cell function. Recent work strengthens the implication of Elongator dependent tRNA modifications in sustaining metastasis and uncovered potential regulatory inputs into the modifier complex. Further work will be required to determine whether drug-based interference with Elongator function or regulation opens new approaches to cancer therapy.

## Figures and Tables

**Table 1:** Transfer RNA (tRNA) modification genes linked to human disease.

Disease	Genes	Modification
Familial dysautonomia ^1^ [21,22,23]	*IKBKAP*	mcm^5^(s^2^)U_34_, ncm^5^U_34_, ncm^5^Um_34_
Intellectual disability ^1^ [24,25]	*ELP2*	mcm^5^(s^2^)U_34_, ncm^5^U_34_, ncm^5^Um_34_
Amyotrophic lateral sclerosis ^1^ [26,27]	*ELP3*	mcm^5^(s^2^)U_34_, ncm^5^U_34_, ncm^5^Um_34_
Breast-, bladder-, colorectal-, cervix- and testicular cancer^1^ [28]	hTRM9L *	mcm^5^(s^2^)U_34_ (? *)
Urothelial cancer ^2^ [29]	*ALKBH8*	mcm^5^(s^2^)U_34_
Asthma ^1^ [30]	*IKBKAP*	mcm^5^(s^2^)U_34_, ncm^5^U_34_, ncm^5^Um_34_
Melanoma ^2,3^ [31,32]	*ELP1, 3, 5, 6, CTU1/2*	mcm^5^(s^2^)U_34_, ncm^5^U_34_, ncm^5^Um_34_
Invasive breast cancer ^2,3^ [33]	*ELP3, CTU1/2*	mcm^5^(s^2^)U_34_, ncm^5^U_34_, ncm^5^Um_34_
X-linked mental retardation ^1^ [34]	*FTSJ1*	Cm_32_, Gm_34_, yW_37_, ncm^5^Um_34_
MELAS (mitochondrial encephalomyopathy, lactic acidosis and stroke-like episodes) ^1^ [11,35,36,37]	Mt-tRNA ^Leu^_UAA_´, *MTO1, GTPBP3*	τm^5^U_34_ (mito)
MERRF (myoclonus epilepsy with ragged-red fibers) ^1^ [11,38]	Mt-tRNA ^Lys^_UUU,_ *MTO1, GTPBP3, MTU1*	τm^5^s^2^U_34_ (mito)
Deafness associated with rRNA A1555G mutation ^1^ [39]	*MTU1*	s^2^U (mito)
Acute infantile liver failure^1^ [40]	*MTU1*	s^2^u (mito)
Neurodegeneration, Galloway-Mowat syndrome^1^ [41,42]	*YRDC, KEOPS, OSGEPL1*	t^6^A_37_
MERRF-like syndrome ^1^ [9]	*YRDC, KEOPS, OSGEPL1*	t^6^A_37_
Type 2 diabetes ^1^ [43,44]	*CDKAL1*	ms^2^t^6^A_37_
Breast cancer ^2^ [45]	*TRMT12*	wybutosine_37_
Intellectual disability ^1^ [46,47]	*PUS3*	Ψ_38/39_
Intellectual disability ^1^, Microcephaly ^1^, aggressive behavior ^1^ [48]	*PUS7*	Ψ_13/35_
Intellectual disability ^1^ [49]	*NSUN2*	m^5^C_34,48,49_
Dubowitz-like syndrome ^1^ [50]	*NSUN2*	m^5^C_34,48,49_
Noonan-like syndrome ^1^ [51]	*NSUN2*	m^5^C_34,48,49_
Skin-, breast- and colorectal cancer ^2,3^ [52,53,54]	*NSUN2*	m^5^C_34,48,49_
Intellectual disability ^1^ [55]	*ADAT3*	I_34_
Encephalopathy and myoclonic epilepsy ^1^ [56]	*TRIT1*	i^6^A/ms^2^i^6^A_37_
Lung- and breast cancer ^1^ [57,58,59]	*TRIT1*	i^6^A/ms^2^i^6^A_37_
Intellectual disability ^1^ [24]	*TRMT1*	m^2,2^G_26_
Primordial dwarfism ^1^ [60]	*METTL1/WDR4, TRM82*	m^7^G_46_
PEPS ^1^ (Partial epilepsy with pericentral spikes) [61]	*TRMT44*	Um_44_
Microcephaly ^1^ [62,63]	*TRMT10A*	m^1^G_9_
Intellectual disability and early onset diabetes ^1^ [64,65], epilepsy ^1^ [65]	*TRMT10A*	m^1^G_9_
Breast cancer ^2^ [66]	*TRMT2A*	m^5^U_54_
Mitochondrial Myopathy and Sideroblastic Anemia ^1^ (MLASA) [67]	*PUS1*	Ψ_multiple_ (mito)

^1^ Disease associated with mutation or downregulation of modification gene; ^2^ Disease associated with upregulation of modification gene; ^3^: Depletion of modification enzyme impaired tumorigenicity or cancer cell viability; * A tRNA methyltransferase activity of hTRM9L has not yet been demonstrated. In cases when modification genes are involved in formation of specific parts of complex modifications, this is indicated by underlining the relevant part. Modifications are abbreviated according to [14]. Mito: modification in mitochondrial tRNA.

**Table 2:** Yeast and human genes of the Elongator and ubiquitin related modifier 1 (Urm1) pathways. See text for references. Parts of the modification in which individual genes are involved are underlined.

Yeast Gene	Human Orthologs/Synonym	Modifications
*ELP1*	*ELP1/IKAP*	mcm^5^s^2^U; mcm^5^U; ncm^5^U; ncm^5^Um
*ELP2*	*ELP2*	mcm^5^s^2^U; mcm^5^U; ncm^5^U; ncm^5^Um
*ELP3*	*ELP3*	mcm^5^s^2^U; mcm^5^U; ncm^5^U; ncm^5^Um
*ELP4*	*ELP4*	mcm^5^s^2^U; mcm^5^U; ncm^5^U; ncm^5^Um
*ELP5*	*ELP5*	mcm^5^s^2^U; mcm^5^U; ncm^5^U; ncm^5^Um
*ELP6*	*ELP6*	mcm^5^s^2^U; mcm^5^U; ncm^5^U; ncm^5^Um
*TRM9*	*ALKBH8, hTRM9L* *	mcm^5^s^2^U; mcm^5^U
*TRM112*	*TRMT112*	mcm^5^s^2^U; mcm^5^U
*NFS1*	*NFS1*	mcm^5^s^2^U
*TUM1*	*TUM1*	mcm^5^s ^2^U
*URM1*	*URM1*	mcm^5^s^2^U
*UBA4*	*UBA4*	mcm^5^s^2^U
*NCS2*	*CTU1*	mcm^5^s^2^U
*NCS6*	*CTU2*	mcm^5^s^2^U

* A direct demonstration of tRNA methyltransferase activity is missing for hTRM9L. Modifications are abbreviated according to [14].

**Table 3:** Diphthamide synthesis genes and defects linked to disease syndromes in higher eukaryotes.

Gene	Elongation factor 2 (EF2) Modification	xm ^5^U_34_ Modification	Species	Disease/Syndrome
*DPH1*	absent	present	Human	Lung cancer [205]
				Breast cancer [206,207]
				Brain tumors [208]
				Ovarian cancer [209,210]
				Colorectal cancer [211]
				Intellectual disability and craniofacial abnormalities [212,213]
				Miller-Dieker syndrome (MDS) [214]
				Airway obstruction and external genital abnormalities [215]
			Mouse	Embryonic lethal, cell proliferation defect, edema, polydactyly [216] Embryonic jaw shortening, cleft palate [214]
			Fruit fly	Failure of intestinal stem cell proliferation [217]
*KTI11/DPH3*	absent	absent	Chinese hamster	Reduced life span hunder [218]
			Mouse	Necrosis, apoptosis and defects in development of placenta [219]
*DPH4*	absent	present	Mouse	Neuronal underdevelopment, impaired growth and polydactyly [220]
*DPH5*	absent	present	Fruit fly	Intestinal stem cell defect [217]
